# Intervention effects of long-term exercise on executive function in children and adolescents with attention-deficit hyperactivity disorder: a three-level meta-analysis of randomised controlled trials

**DOI:** 10.7189/jogh.16.04233

**Published:** 2026-07-03

**Authors:** Haiyan Wang, Jilan Zhou, Lei Zhao

**Affiliations:** 1 Department of Physical Education, China University of Petroleum (East China), Qingdao, China; 2 School of Health Sciences, University Sains Malaysia, Kelantan, Malaysia; 3 Physical Education Department, Qingdao Huanghai University, Qingdao, China

## Abstract

**Background:**

Attention-deficit hyperactivity disorder (ADHD) is a common neurodevelopmental disorder in children and is often associated with executive function impairments. We aimed to examine the effects of long-term exercise on executive function in children and adolescents with ADHD using a three-level meta-analysis.

**Methods:**

We searched five databases (Embase, The Cochrane Library, PubMed, Web of Science, and China National Knowledge Infrastructure) from inception to 7 June 2025, for randomised controlled trials on long-term exercise and executive function in children and adolescents with ADHD. We used ROB 2 to assess the risk of bias. We used a three-level meta-analysis with restricted maximum likelihood as the primary model to synthesise non-independent effect sizes. We conducted influence analysis to identify influential cases, and sensitivity analyses using an aggregated two-level random-effects model and robust variance estimation. We assessed evidence quality with GRADEpro.

**Results:**

We included 15 studies. We derived the primary estimate from the outlier-removed three-level model because three influential effect sizes were identified, and we conducted subsequent secondary analyses on the same data set. The results showed that long-term exercise may improve executive function in children and adolescents with ADHD (Hedges’ g = −0.52; 95% confidence interval = −0.87, −0.18; *P* = 0.003), with low-certainty evidence. Sensitivity analyses yielded effects in a similar direction and of comparable magnitude. Intervention duration (F = 4.72; *P* = 0.033) and age (F = 5.36; *P* = 0.023) were identified as moderating factors for the improvement of executive function in children and adolescents with ADHD through long-term exercise.

**Conclusions:**

Long-term exercise may benefit executive function in children and adolescents with ADHD, although the certainty of the evidence was low. Findings on age and intervention duration should be interpreted cautiously because some subgroup strata included only a small number of effect sizes.

**Registration:**

PROSPERO: CRD42024627175.

Attention-deficit hyperactivity disorder (ADHD) is a prevalent neurodevelopmental disorder in children, characterised by core symptoms of hyperactivity and impulsivity that are incongruent with the individual's developmental level, both in terms of physical and cognitive growth [1]. Approximately 5% of children worldwide are affected by ADHD, with the prevalence showing an increasing trend [2]. The core symptoms of ADHD are closely associated with lower levels of executive function [3], which are primarily manifested in impaired inhibition, working memory, and cognitive flexibility among children and adolescents with ADHD [4]. These impairments not only affect motor skills in ADHD adolescents but also have an impact on their social achievements in adulthood [5,6].

Long-term pharmacological treatment may lead to the development of tolerance and an increased risk of adverse side effects [7,8]. Exercise, due to its high practicality and minimal side effects, has been applied as an intervention to improve executive function in children and adolescents with ADHD [9]. Ziereis *et al.* conducted a 12-week comprehensive exercise intervention for children with ADHD, and the results indicated that exercise can improve working memory in these children [10]. Benzing *et al.* implemented an eight-week Exergaming programme for children with ADHD, which showed significant improvements only in inhibition and cognitive shifting reaction times, with no improvements observed in other aspects of executive function [5]. These findings suggest that the efficacy of long-term exercise in improving executive function in children and adolescents with ADHD remains controversial.

Currently, several studies have used meta-analysis to explore the effects of exercise on executive function in children and adolescents with ADHD, but the results remain controversial. Yang *et al.* applied traditional meta-analysis methods and found that exercise could improve inhibition and cognitive shifting in children with ADHD, but did not affect working memory [11]. Dastamooz *et al.* also indicated that exercise can improve inhibition, cognitive flexibility, and attention in children and adolescents with ADHD, but did not show improvements in working memory [12]. In contrast, Sun *et al.* used traditional meta-analysis and found that exercise could improve executive function in children with ADHD [13]. Similarly, Liang *et al.* reported that exercise could enhance inhibition, working memory, and cognitive flexibility in children and adolescents with ADHD [14].

Because we needed to consider multiple executive function indicators, several effect sizes meeting the inclusion criteria are likely to be reported within the same study. Additionally, considering the assumption of independence between effect sizes in traditional meta-analysis and the potential impact of correlations between effect sizes on the combined effect size, we intend to depart from traditional meta-analysis and adopt a three-level meta-analysis approach. A three-level meta-analysis refers to a model where variance originates from three levels: sampling variance (Level 1), within-study variance (Level 2), and between-study variance (Level 3) [15]. By accounting for these three components of variance, a three-level meta-analysis allows for the extraction of all eligible effect sizes from the same study, reducing the issue of statistical power loss due to information loss. Furthermore, it can avoid the exaggeration of results caused by the correlation between effect sizes in traditional meta-analyses [16].

Therefore, we employed a three-level meta-analysis to include all relevant effect sizes and systematically evaluate the effects of long-term exercise on executive function in children and adolescents with ADHD. In addition, we aimed to explore whether factors such as age, executive function subcomponents, exercise type, session duration, intervention duration, and exercise frequency may moderate these effects, seeking to inform future research and clinical practice.

## METHODS

We conducted this meta-analysis following the PRISMA 2020 guidelines [17], ensuring methodological rigour and transparency (Checklist S1 in the **Online Supplementary Document**). We prospectively registered the study protocol in the PROSPERO database (registration ID: CRD42024627175).

### Search strategy

The researchers independently conducted computer-based searches across five databases: China National Knowledge Infrastructure, PubMed, The Cochrane Library, Embase, and Web of Science, for randomised controlled trials (RCTs) evaluating long-term exercise interventions targeting executive function in children and adolescents with ADHD. The search strategy combined terms related to exercise, physical activity, attention-deficit/hyperactivity disorder, children and adolescents, and executive function. Subject headings and free-text terms were combined using Boolean operators. The search period extended from the inception of each database up to 7 June 2025. (Appendix S1 in the **Online Supplementary Document**).

### Eligibility criteria

We followed the Population, Intervention, Comparison, Outcomes, and Study criteria when selecting eligible studies:

− Population (P): Included participants were children and adolescents aged 6–18 years with a formal diagnosis of ADHD. Diagnostic confirmation was based on standardised instruments such as the Diagnostic and Statistical Manual of Mental Disorders (DSM)-4, DSM-5, DSM-4-TR, and DSM-10, or clinical evaluation by a qualified medical professional.
− Intervention: The intervention group participated in structured exercise interventions lasting ≥6 weeks. We used the term ‘long-term exercise’ operationally to refer to sustained exercise interventions of ≥6 weeks, to distinguish them from acute or very short-term exercise effects. We adopted this threshold for study selection and analysis, rather than as a universal definition of long-term exercise. Given the substantial variation in intervention duration across studies, we further categorised intervention duration as 6–12 weeks and >12 weeks for moderator analyses. No restrictions were placed on exercise modality, and eligible interventions included aerobic training, resistance exercise, and other structured physical activity programmes [18].
− Comparison: Control groups did not participate in the target exercise intervention and received usual care, medication, sedentary activities, health education, wait-list control, or daily-life conditions, depending on the study design.
− Outcomes: The primary outcomes assessed were core components of executive functioning, including inhibitory control, working memory, and cognitive flexibility.
− Study Design: Only RCTs were included in the analysis.

We excluded studies in which the intervention involved physical activity but consisted of only a single exercise session or had a total duration of less than six weeks; studies for which outcome data required for effect size estimation were unavailable, even after contacting the original authors; publications not written in either English or Chinese; articles that had not undergone peer review, including review papers, conference abstracts, and other non-primary sources.

### Study selection

The researchers imported the retrieved studies into EndNote X9, initially removing duplicates and performing a preliminary screening based on titles and abstracts. Subsequently, we reviewed the full texts of the studies that passed the initial screening. Finally, we included the studies that met the inclusion criteria in the meta-analysis.

### Data extraction and coding strategy

Two researchers (HYW and LZ) independently extracted data using a pre-designed Microsoft Excel spreadsheet. In cases of disagreement, a third researcher (JLZ) was consulted until a consensus was reached. Extracted data included general study characteristics (*e.g.* author, publication year, sample size, and participant age), exercise intervention parameters (*e.g.* type of exercise, intensity, frequency, session duration, and intervention duration), and outcome measures (*e.g.* reaction time and accuracy in the Stroop task). Outcome data were collected at three time points: pre-intervention and post-intervention. If study information was unclear or data were not readily extractable, we contacted the corresponding author via email.

We categorised executive function into three core components: inhibition, working memory, and cognitive flexibility. In addition, we coded executive function outcomes according to their measurement properties as either accuracy/error-based or latency-based outcomes. This classification was based on the specific reported metric rather than the task name itself. We coded accuracy, correct responses, omission/nonresponse indices, and error counts as accuracy/error-based outcomes, and reaction time, completion time, and other time-related performance indices as latency-based outcomes. When multiple indices were reported within the same task, we extracted them as separate effect sizes rather than combined into a single composite outcome, and assessed their statistical dependency using the three-level meta-analytic model. We classified exercise type as either closed- or open-skill. Closed-skill exercises are performed in stable and predictable environments, with minimal influence from external variability; movement patterns tend to be consistent and repetitive. In contrast, open-skill exercises take place in dynamic and unpredictable settings, requiring participants to rapidly perceive and respond to changing stimuli (*e.g.* opponents, objects, or environmental cues), thereby demanding higher levels of perceptual, cognitive, and motor adaptability [18]. We dichotomised session duration as ≤60 and >60 minutes and grouped intervention duration into 6–12 and ≥12 weeks [19]. We coded exercise frequency as either 2–3 or 4–5 sessions per week. We stratified participants' age into two groups: <12 and ≥12 years. We conducted moderator analyses to explore potential sources of heterogeneity and interpreted them cautiously, particularly for subgroup strata supported by a small number of effect sizes.

### Risk of bias and quality of evidence assessment

The two researchers (HYZ and LZ) independently assessed the risk of bias using the revised Cochrane Risk-of-Bias tool for Randomised Trials (ROB 2). In case of any disagreements, a third researcher (JLZ) was involved in the discussion until a consensus was reached. ROB 2 primarily assesses the risk of bias through the following domains: randomisation process, deviations from intended interventions, missing outcome data, measurement of the outcome, and selection of the reported result.

We used GRADEpro software, version 3.6.1 (McMaster University and Evidence Prime, Hamilton, Ontario, Canada) to assess the quality of outcome evidence. We rated the evidence based on five domains: ‘risk of bias’, ‘inconsistency’, ‘indirectness’, ‘imprecision’ and ‘publication bias’. We categorised the overall quality of evidence as ‘high’, ‘moderate’, ‘low’ or ‘very low’.

### Data analysis

Due to the necessity of including multiple effect sizes from the same study, traditional meta-analytic methods were insufficient for our research. Therefore, we conducted a three-level meta-analysis.

We estimated variance components at all three levels, and applied the one-tailed log-likelihood ratio tests (LRTs) to assess the statistical significance of variance at Levels 2 and 3 [20]. We pooled the effect sizes using the restricted maximum likelihood estimator. We calculated standardised mean differences (Hedges’ g (g)) as the measure of effect size. For studies reporting both baseline and post-intervention data, we primarily calculated the effect sizes based on change scores. We defined the mean change score as the post-intervention mean minus the baseline mean. Because most included studies did not directly report the standard deviation (SD) of the change score, we derived the SD of the change score from the baseline and post-intervention SD using the following formula:



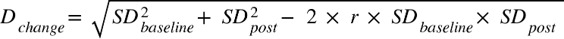



where r represents the correlation coefficient between baseline and post-intervention measurements. When this correlation was not reported in the original studies, we assumed a common correlation coefficient of r = 0.50 for both the intervention and control groups to ensure consistency in the calculation of effect sizes across studies. If only standard errors (SEs) were reported, we first converted them into SDs using the formula:



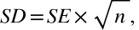



after which, we calculated the SD of the change score. We used change scores, rather than post-intervention values alone, as the primary basis for effect-size calculation because most ADHD RCTs we included had relatively small sample sizes, and some degree of baseline imbalance may still have existed despite randomisation. In this context, change scores make use of both baseline and post-intervention information and were therefore considered a more appropriate basis for the primary analysis. In addition, most included studies did not report baseline-adjusted effect estimates derived from analysis of covariance. Therefore, we adopted change scores as a uniform strategy for effect-size calculation.

To ensure a consistent interpretation of effect sizes across executive function outcomes, we harmonised the direction of all measures before analysis. For outcomes in which lower values indicated better performance (*e.g.* reaction time, error counts, interference scores), we retained effect sizes in their original direction. For outcomes in which higher values indicated better performance (*e.g.* accuracy, number of correct responses), we revised the direction of the effect size when necessary. Accordingly, a negative g consistently indicated better executive function performance in the exercise group relative to the control group. We reported effect sizes as g with 95% confidence intervals (CI) and prediction intervals (PI). Consistent with prior literature, we categorised effect sizes as: small (g < 0.20), small to moderate (g = 0.20–0.49), moderate (g = 0.50–0.79), and large (g ≥ 0.80) [21].

To mitigate the influence of outliers, we employed influence analysis to identify potential influential cases [22]. Additionally, we conducted subgroup analyses; when ≥ 2 variables served as moderators, we applied a multiple moderator model [16]. We assessed publication bias through visual inspection of funnel plots and Egger’s regression test. We considered evidence of publication bias to be present if the funnel plot exhibited substantial asymmetry and the Egger test yielded a *P*-value of <0.05. In such cases, we employed the trim-and-fill method to adjust for potential bias [23].

In addition, we conducted sensitivity analyses to evaluate the robustness of the findings to alternative approaches for handling dependent effect sizes. Specifically, we compared the primary three-level model with a conventional two-level random-effects model based on one aggregated effect size per study and a robust variance estimation (RVE) approach using cluster-robust inference.

We used the metafor package, version 3.2-2 in *R*, version 4.4.1 (R Core Team, Vienna, Austria), employing the code developed by Assink and Wibbelink [16,24] for the three-level meta-analysis.

## RESULTS

### Literature search

**Figure 1:**
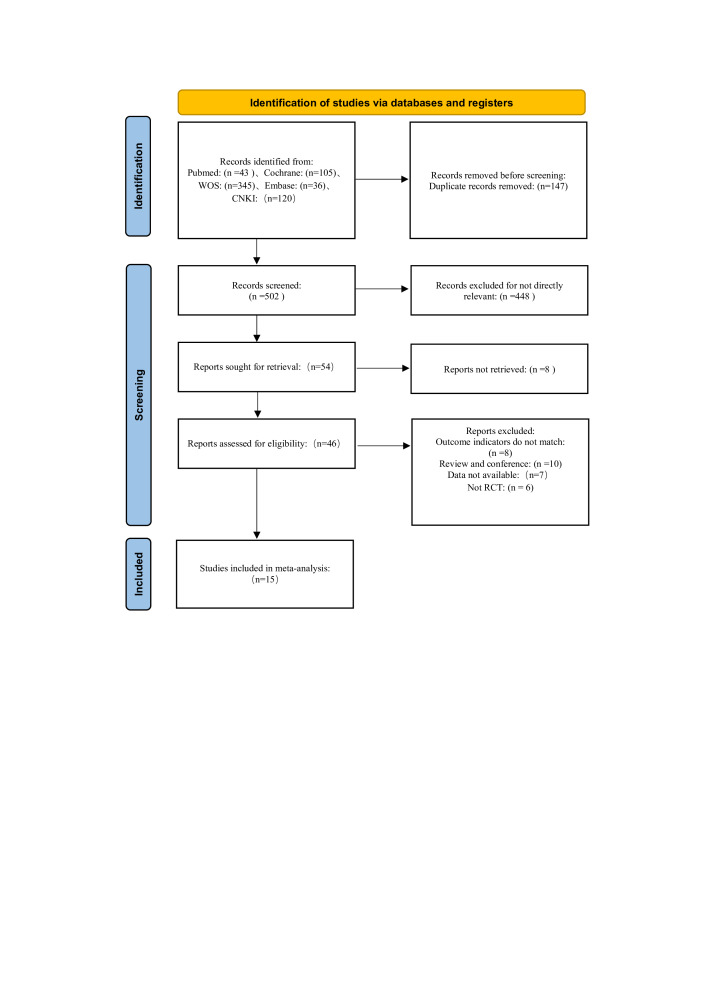
Flowchart of the study selection process. CNKI – China National Knowledge Infrastructure, RCT – randomised controlled trial, WOS – Web of Science.

We retrieved 649 articles from five databases (**Figure 1**). After removing 147 duplicates, we screened 502 articles based on titles and abstracts, excluding 448 irrelevant articles. We downloaded the full texts of 54 articles, and eight articles could not be retrieved. We assessed the remaining 46 articles through full-text review. We excluded eight studies due to irrelevant data, ten were conference proceedings or review articles, seven could not provide extractable data, and six were non-RCTs. Ultimately, we included 15 studies [5,25–38].

### Characteristics of included studies

We included 15 studies, originating from Switzerland, the USA, China, Tunisia, Netherlands, the Republic of Korea, Iran, and Brazil (**Table 1**). The studies mostly used ICD-10 and DSM-5 for diagnostic criteria. The interventions involved a range of exercise modalities, including exergaming, indoor cycling, and judo training, with most interventions lasting 8–12 weeks. Exercise frequency was typically 2–3 sessions per week, and session duration was generally 30–60 minutes. Outcome measures included the Stroop test, the Wisconsin Card Sorting Test, and other executive function tasks.

**Table 1:** Characteristics of included studies

Study author (year)	Country	Sample size	Age (years), x̄ (SD)	Diagnostic mode	Intervention content	Outcome
Benzing and Schmidt (2019) [5]	Switzerland	E: 28, C: 23	E: 10.46 (1.30), C: 10.39 (1.44)	ICD-10	E: Exergaming intervention, 8 weeks, 3 times a week, 30 min; C: Waiting-list control group	Modified Simon Task, modified Flanker task, modified version of the colour span backwards task
Bustamante *et al.* (2016) [25]	USA	E: 16, C: 19	E: 8.7 (2), C: 9.4 (2.2)	DSM-IV	E: Physical activity, 10 weeks, 5 times a week, 60 min; C: Sedentary play	STOPIT task, The Automated Working Memory Assessment System
Chang *et al.* (2022) [26]	China	E1: 16, E2: 16, C: 16	E1: 8.31 (1.3), E2: 8.38 (1.2), C: 8.38 (1.31)	DSM-5	E1: Simulated table tennis, 12 weeks, 5 times a week, 60 min; E2: Actual table tennis; C: Sedentary play	Stroop Test, Wisconsin Card Sorting Test
Chen *et al.* (2022) [27]	China	E: 32, C: 32	E: 8.37 (1.68), C: 7.89 (2.13)	DSM-5	E: Indoor bike, 12 weeks, 3 times a week, 20 min; C: Sedentary play	Stroop, Size odd and even, N-back
Choi *et al.* (2015) [28]	China	E: 13, C: 17	E: 15.9 (1.2), C: 16 (1.2)	DSM-IV	E: Aerobic exercises, 6 weeks, 3 times a week, 90 min, 60% HRmax; C: Health education	Wisconsin Card Sorting Test
Geladé *et al.* (2017) [29]	Netherlands	E: 37, C: 36	E: 9.8 (1.96), C: 9.11 (1.26)	DSM-IV-TR	E: Aerobic exercises, 10–12 weeks, 3 times a week, 45 min, 70–100% HRmax; C: Medication	Stop-signal task, Visual spatial working memory task
Kadri *et al.* (2019) [30]	Tunisia	E: 20, C: 20	E: 14.5 (3.5), C: 14.2 (3.0)	Unspecified	E: Taekwondo, 1.5 y, 2 times a week, 50 min; C: Daily life	Stroop
Lee *et al.* (2017) [31]	Republic of Korea	E: 6, C: 6	E: 8.83 (0.98) C: 8.83 (0.98)	DSM-IV	E: Physical activity, 12 weeks, 3 times a week, 60 min, 45–75%HR reserve; C: Medication	Stroop
Liang *et al.* (2022) [32]	China	E: 40, C: 40	E: 8.37 (1.42), C: 8.29 (1.27)	DSM-5	E: Aerobic and neurocognitive exercise programme, 12 weeks, 3 times a week, 60 min; C: Wait-list control group	Flanker, Trail Making Test
Liu and Yang (2018) [33]	China	E: 32, C: 32	8–12	DSM-IV	E: Directional movement, 3 times a week, 35 min, 14 weeks, 60–70% HRmax; C: Daily life	Corsi blocks the click task
Ludyga *et al.* (2022) [34]	Switzerland	E: 29, C: 28	8–12	DSM-5	E: Judo training, 2 times a week, 60 min, 12 weeks; C: Wait-list control group	Change Detection paradigm
Memarmoghaddam *et al.* (2016) [35]	Iran	E: 19, C: 17	E: 8.31 (1.29), C: 8.29 (1.31)	DSM-IV	E: Physical activity, 3 times a week, 90 min, 8 weeks; C: No intervention was given	Stroop, Go NoGo
Pan *et al.* (2016) [36]	China	E: 16, C: 16	E: 8.93 (1.49), C: 8.87 (1.56)	DSM-IV	E: Racket-sport intervention, 2 times a week, 70 min, 12 weeks; C: Daily life	Stroop
Silva *et al.* (2020) [37]	Brazil	E: 10, C: 10	E: 12 (2), C: 12 (1)	DSM-IV	E: Swimming, 2 times a week, 45 min, 8 weeks; C: untrained group	Trail Making Test
Song *et al.* (2023) [38]	China	E: 8, C: 8	8–12	DSM-IV	E: Football, 5 times a week, 60 min, 6 weeks; C: Daily life	Stroop, Complex Figure Test, Trail-making test

C – control group, DSM – Diagnostic and Statistical Manual of Mental Disorders, E – experimental group, E1 – experimental group 1, E2 – experimental group 2, HR – heart rate, ICD – International Statistical Classification of Diseases and Related Health Problems, SD – standard deviation, x̄ – mean

### Risk of bias

Of the 15 studies, six studies were rated as low risk for the ‘randomisation process’, 13 studies were rated as low risk for ‘deviations from the intended interventions’ and ‘missing outcome data’, and 14 studies were rated as low risk for ‘measurement of the outcome’ (Figure S1 in the **Online Supplementary Document**). Ten studies were rated as low risk for ‘selection of the reported result’.’ In the ‘overall’ assessment, four studies were classified as low risk, four studies as high risk, and seven studies raised ‘Some concerns’.

### Meta-analysis results

**Figure 2:**
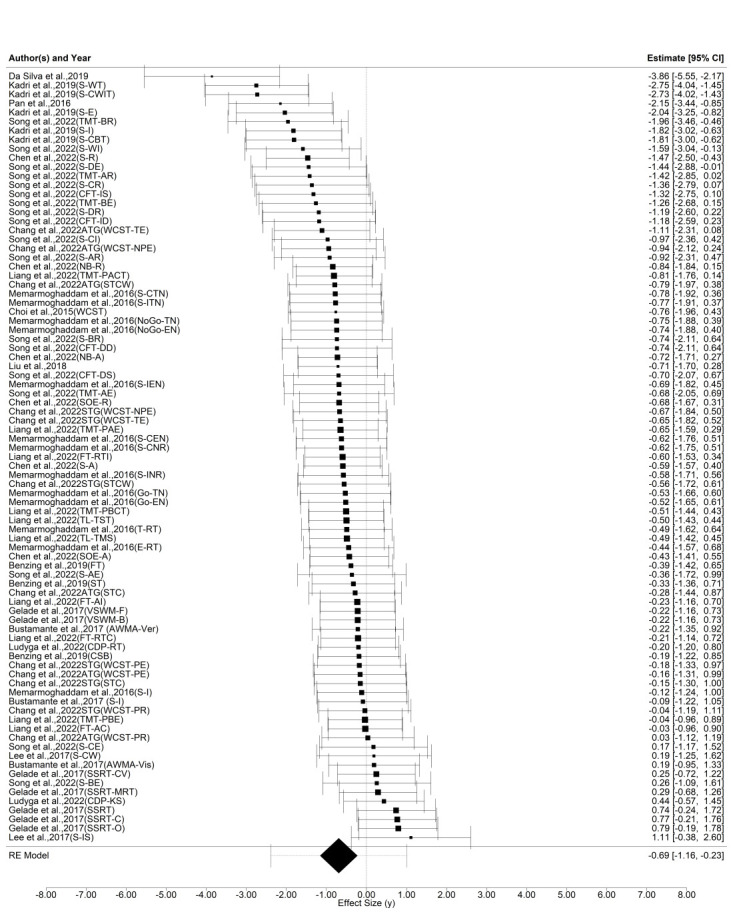
The intervention effect of long-term exercise on executive function in children and adolescents with ADHD. ATG – actual training group, AWMA-Ver – Automated Working Memory Assessment System-Verbal, AWMA-Vis – Automated Working Memory Assessment System-Visuospatial, CDP-KS – Change Detection Paradigm-K score, CDP-RT – Change Detection Paradigm-reaction time, CFT-DD – Complex Figure Test-delayed detail, CFT-DS – Complex Figure Test-delayed structure, CFT-ID – Complex Figure Test-immediate detail, CFT-IS – Complex Figure Test-immediate structure, CI – confidence interval, CSB – colour span backwards, E-RT – error reaction time, FT – Flanker Task, FT-AC – Flanker Task-Accuracy Congruent, FT-AI – Flanker Task-Accuracy Incongruent, FT-RTC – Flanker Task-Reaction Time Congruent, FT-RTI – Flanker Task-Reaction Time Incongruent, Go-EN – Go-Error number, Go-TN – Go-True number, NB-A – N-Back-Accuracy, NB-R – N-Back-Reaction time, NoGo-EN – No-Go-Error number, NoGo-TN – No-Go-True number, S-A – Stroop-accuracy, S-AE – Stroop A-card errors, S-AR – Stroop A-card time, S-BE – Stroop B-card errors, S-BR – Stroop B-card time, S-CE – Stroop C-card errors, S-CI – Stroop colour interference time, S-CR – Stroop C-card time, S-DE – Stroop D-card errors, S-DR – Stroop D-card time, S-WI – Stroop word-meaning interference time, S-CBT – Stroop-Colour Block Test, S-CEN – Stroop-Consistent Error number, S-CNR – Stroop-Consistent No response, S-CTN – Stroop-Consistent True number, S-CW – Stroop-Colour Word, S-CWIT – Stroop-Colour-Word Interference Test, S-E – Stroop-Error, S-I – Stroop-Interference, S-IEN – Stroop-Inconsistent Error number, S-INR – Stroop-Inconsistent No response, S-IS – Stroop-Interference Score, S-ITN – Stroop-Inconsistent True number, S-R – Stroop-reaction time, S-WT – Stroop-Word Test, SOE-A – size odd and even-accuracy, SOE-R – size odd and even-reaction time, SSRT – Stop-signal reaction time, SSRT-C – Stop-signal reaction time-commission, SSRT-CV – Stop-signal reaction time-coefficient of variation, SSRT-MRT – Stop-signal reaction time-response speed, SSRT-O – Stop-signal reaction time-omission errors, ST – Simon Task, STC – Stroop Test Colour, STCW – Stroop Test Colour-Word, STG – simulated training group, TL-TMS – Tower of London-total move scores, TL-TST – Tower of London-total solution time, TMT-AE – Trail Making Test-part A errors, TMT-AR – Trail Making Test-part A time, TMT-BE – Trail Making Test-part B errors, TMT-BR – Trail Making Test-part B time, T-RT – true reaction time, TMT-PACT – Trail Making Test-part A completion time, TMT-PAE – Trail Making Test-part A errors, TMT-PBCT – Trail Making Test-part B completion time, TMT-PBE – Trail Making Test-part B errors, VSWM-B – visual spatial working memory task-backward, VSWM-F – visual spatial working memory task-forward, WCST-NPE – Wisconsin Card Sorting Test-Non-Perseverative Errors, WCST-PE – Wisconsin Card Sorting Test-Perseverative Errors, WCST-PR – Wisconsin Card Sorting Test-Perseverative Responses, WCST-TE – Wisconsin Card Sorting Test-Total Errors.

Our three-level meta-analysis included 85 effect sizes to examine the impact of long-term exercise on executive function in children and adolescents with ADHD (**Figure 2**). The three-level model indicated that long-term exercise significantly improved executive function in children and adolescents with ADHD (g = −0.69; 95% CI = −1.16, −0.23; *P* = 0.004; 95% PI = −2.39, 1.01). The two-level random effects model revealed that long-term exercise also improved executive function in children and adolescents (g = −0.57; 95% CI = −0.72, −0.42; *P* < 0.001; 95% PI = −1.33, 0.18).

### Influence analysis

We conducted an influence analysis to examine the presence of outliers (Figure S2 in the **Online Supplementary Document**). We identified three outliers [30,37]. After removing the outliers, we merged 82 effect sizes. The three-level model showed that long-term exercise significantly improved executive function in children and adolescents with ADHD (g = −0.52; 95% CI = −0.87, −0.18; *P* = 0.003; 95% PI = −1.68, 0.63). The two-level random effects model indicated that long-term exercise also improved executive function in children and adolescents (g = −0.49; 95% CI = −0.62, −0.36; *P* < 0.001; 95% PI = −0.82, −0.17). Because the influence analysis identified potentially influential cases, we conducted subsequent subgroup analyses and publication bias analyses on the outlier-removed data set to reduce the potential impact of these cases on secondary analyses (Figure S3 in the **Online Supplementary Document**).

### Sensitivity analyses

To evaluate the robustness of the main findings to alternative approaches for handling dependent effect sizes, we conducted two additional sensitivity analyses. First, we aggregated multiple effect sizes within each study into a single study-level effect size and performed a conventional two-level random-effects meta-analysis. This aggregated two-level model, based on 15 study-level effect sizes, showed that long-term exercise significantly improved executive function in children and adolescents with ADHD (g = −0.68; 95% CI = −1.18, −0.17; *P* = 0.012; 95% PI = −2.37, 1.02).

Second, we conducted an RVE analysis based on the three-level model to account for within-study dependence using cluster-robust inference. The RVE model also yielded a significant pooled effect (g = −0.69; 95% CI = −1.19, −0.19; *P* = 0.010). Taken together, the sensitivity analyses yielded effect estimates that were similar in direction and magnitude to those of the primary three-level model, providing supportive evidence for the stability of the findings across alternative methods for handling effect-size dependence.

Because we conducted subsequent secondary analyses on the outlier-removed data set, we further repeated these sensitivity analyses after excluding the identified outliers. After outlier removal, the aggregated two-level random-effects model, based on 14 study-level effect sizes, still showed a significant pooled effect (g = −0.50; 95% CI = −0.87, −0.13; *P* = 0.012; 95% PI = −1.50, 0.51). Likewise, the corresponding RVE analysis based on the outlier-removed three-level model also remained significant (g = −0.52; 95% CI = −0.90, −0.15; *P* = 0.010). These results further support the robustness of the main findings after excluding influential cases.

### Heterogeneity analysis

The LRT indicated that within-study variance (Level 2) was not significant (LRT = 0.00; *P* > 0.49), suggesting that the three-level model was not superior to the two-level model. Between-study variance (Level 3) was significant (LRT = 25.46; *P* < 0.001), indicating that the three-level model outperformed the two-level model. In the total variance, sampling variance (Level 1) accounted for 51.32%, within-study variance (Level 2) accounted for 2.20%, and between-study variance (Level 3) accounted for 48.68%.

### Moderator analyses

We conducted moderator analyses as exploratory analyses to examine potential sources of heterogeneity; therefore, findings, particularly those based on a small number of effect sizes, should be interpreted cautiously. We conducted moderation effect tests for subdomains of executive function, exercise type, session duration, intervention duration, exercise frequency, age, and outcome type (**Table 2**).

**Table 2:** Moderators of the intervention effect of exercise on EF in children and adolescents with ADHD

						Variance	
	**k**	**g (95% CI)**	**F**	**DF**	***P*-value**	**Level 2**	**Level 3**
**EF subdomains**			0.45	2	0.64	1.36	50.87
Inhibitory control	46	−0.45 (−0.84, −0.07)			0.022		
Working memory	16	−0.59 (−1.03, −0.16)			0.008		
Cognitive flexibility	20	−0.59 (−1.04, −0.15)			0.010		
**Exercise type**			0.61	1	0.44	1.18	50.26
Closed-skill	13	−0.22 (−1.07, 0.64)			0.61		
Open-skill	69	−0.59 (−0.97, −0.20)			0.004		
**Intervention duration in weeks**			4.72	1	0.033	2.65	37.46
6 − 12	78	−0.40 (−0.71, −0.09)			0.011		
≥12	4	−1.40 (−2.26, −0.54)			0.002		
**Session duration in minutes**			1.53	1	0.219	1.53	0.22
≤60	67	−0.42 (−0.81, −0.04)			0.032		
>60	15	−1.00 (−1.84, −0.16)			0.021		
**Exercise frequency times per week**			0.00	1	0.99	1.26	52.26
2–3	61	−0.52 (−0.92, −0.13)			0.011		
4–5	21	−0.53 (−1.45, 0.38)			0.251		
**Age in years**			5.36	1	0.023	2.44	35.82
<12	78	−0.40 (−0.70, −0.10)			0.009		
≥12	4	−1.48 (−2.37, −0.60)			0.001		
**Outcome type**			0.66	1	0.42	1.19	45.16
Accuracy/error-based	49	−0.48 (−0.84, −0.11)			0.011		
Latency-based	33	−0.59 (−0.97, −0.21)			0.003		

CI – confidence interval, DF – degrees of freedom, EF – executive function, F – F statistic, g – Hedges’ g, k – number of effect sizes

We categorised executive function into inhibitory control, working memory, and cognitive flexibility, with executive function not identified as a significant moderator (F = 0.45; *P* = 0.64). Exercise was found to improve inhibitory control (g = −0.45; 95% CI = −0.84, −0.07; *P* = 0.022), working memory (g = −0.59; 95% CI = −1.03, −0.16; *P* = 0.008), and cognitive flexibility (g = −0.59; 95% CI = −1.04, −0.15; *P* = 0.010) in children and adolescents with ADHD.

We classified exercise type into closed- and open-skill exercises, with exercise type not identified as a significant moderator (F = 0.61; *P* = 0.44). Closed-skill exercises were not significant in improving executive function in children and adolescents with ADHD (g = −0.22; 95% CI = −1.07, 0.64; *P* = 0.61). However, open-skill exercises were found to improve executive function in children and adolescents with ADHD (g = −0.59; 95% CI = −0.97, −0.20; *P* = 0.004).

We classified intervention duration into ‘6–12 weeks’ and ‘>12 weeks’, with intervention duration identified as a significant moderator (F = 4.72; *P* = 0.033). Both ‘6–12 weeks’ (g = −0.40; 95% CI = −0.71, −0.09; *P* = 0.011) and ‘>12 weeks’ (g = −1.40; 95% CI = −2.26, −0.54; *P* = 0.002) of exercise were found to improve executive function in children and adolescents with ADHD. However, the ‘>12 weeks’ subgroup was represented by only four effect sizes; therefore, this estimate should be interpreted cautiously.

We classified session duration into ‘≤60 minutes’ and ‘>60 minutes’, with session duration not identified as a significant moderator (F = 1.53; *P* = 0.219). Both ‘≤60 minutes’ (g = −0.42; 95% CI = −0.81, −0.04, *P* = 0.032) and ‘>60 minutes’ (g = −1.00; 95% CI = −1.84, −0.16, *P* = 0.021) of exercise were found to improve executive function in children and adolescents with ADHD.

We classified exercise frequency into ‘2–3 times per week’ and ‘4–5 times per week,’ with exercise frequency not identified as a significant moderator (F = 0.00; *P* = 0.99). Exercise ‘2–3 times per week’ improved executive function in children and adolescents with ADHD (g = −0.52; 95% CI = −0.92, −0.13; *P* = 0.011), whereas exercise ‘4–5 times per week’ did not reach statistical significance in improving executive function in children and adolescents with ADHD (g = −0.53; 95% CI = −1.45, 0.38; *P* = 0.251).

We classified age into ‘<12 years’ and ‘≥12 years,’ with age identified as a significant moderator (F = 5.36; *P* = 0.023). Exercise was found to improve executive function in both ‘<12 years’ (g = −0.40; 95% CI = −0.70, −0.10; *P* = 0.009) and ‘≥12 years’ (g = −1.48; 95% CI = −2.37, −0.60; *P* = 0.001) age groups. However, the ‘≥12 years’ subgroup also included only four effect sizes, and thus this estimate should be considered preliminary and interpreted cautiously.

We classified outcome types into ‘accuracy/error-based outcomes’ and ‘latency-based outcomes.’ However, outcome type was not identified as a significant moderator (F = 0.66; *P* = 0.420). Long-term exercise produced significant improvements in both accuracy/error-based (g = −0.48; 95% CI = −0.84, −0.11; *P* = 0.011) and latency-based (g = −0.59; 95% CI = −0.97, −0.21; *P* = 0.003) outcomes.

In addition, we further explored the dose-response relationship between weekly exercise time (session duration × weekly exercise frequency), total exercise time (weekly exercise time × intervention duration), and improvements in executive function. We applied both linear and restricted cubic spline models. The results showed that weekly exercise time was not significantly associated with the intervention effect on executive function in the linear model (β = 0.00; SE = 0.00; *t* = 0.33; *P* = 0.74; 95% CI = −0.00, 0.00), and the overall test of the nonlinear spline model was also not statistically significant (F = 0.37; *P* = 0.778). These findings suggest that no clear linear or nonlinear dose–response relationship was observed between weekly exercise time and improvements in executive function.

By contrast, total exercise time showed a marginally significant trend in the linear model (β = −0.00; SE = 0.00; *t* = −1.85; *P* = 0.068; 95% CI = −0.00, 0.00), suggesting that as total exercise time increased, the effect size tended to decrease, indicating a potentially greater beneficial effect of exercise on executive function. However, the overall test of the nonlinear spline model for total exercise time did not reach statistical significance (F = 1.942; *P* = 0.130), indicating that there is currently insufficient evidence to support a clear nonlinear dose–response relationship between total exercise time and improvements in executive function. Although the third spline term was statistically significant (β = −1.40; *P* = 0.032), the overall spline model was not significant; therefore, the overall model results should be prioritised, and this possible local fluctuation should be interpreted with caution.

### Multiple moderator model

We performed multiple regression analysis to exclude collinearity between significant moderators, using ‘6–12 weeks’ and ‘<12 years’ as reference variables. The omnibus test was significant (F (2,79) = 3.32, *P* = 0.041), suggesting that at least one of the regression coefficients of the moderators significantly deviates from zero (**Table 3**).

**Table 3:** Continuous moderator analyses of long-term exercise on executive function

	k	β (95% CI)	*P*-value
**Intervention duration**			
Intercept		−0.37 (−0.67, −0.07)	0.017
>12 weeks	4	−0.60 (−1.69, 0.48)	0.272
**Age**			
≥12 years	4	−0.73 (−1.86, 0.40)	0.203

CI – confidence interval, k –

### Publication bias

**Figure 3:**
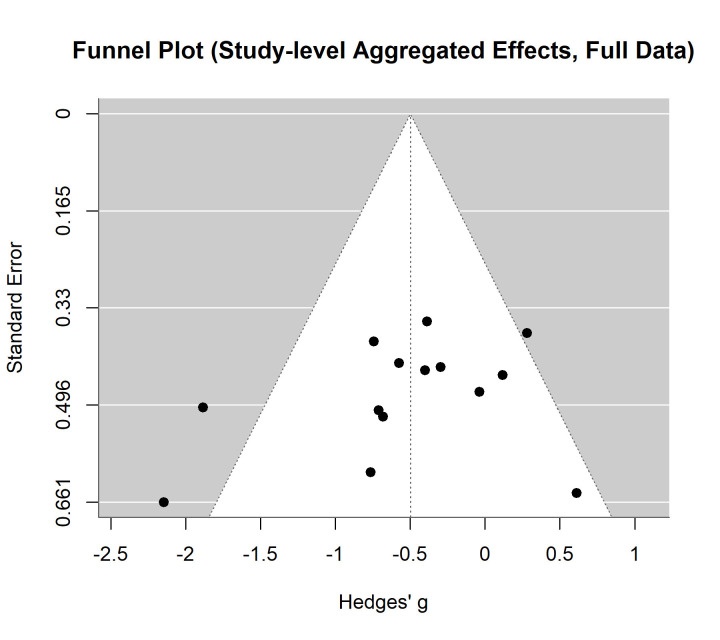
Funnel plot of the effect of long-term exercise on executive function in children and adolescents with ADHD.

We examined funnel-plot asymmetry using study-level aggregated effect sizes (**Figure 3**). Visual inspection of the funnel plot did not reveal marked asymmetry. In addition, Egger’s regression test did not indicate significant funnel-plot asymmetry (*t* = −1.32; *P* = 0.211). Trim-and-fill analysis, used as a supplementary sensitivity analysis, estimated no missing studies, and the adjusted pooled effect remained unchanged (g = −0.50; 95% CI = −0.87, −0.13; *P* = 0.012). These findings suggest that there was no clear evidence of small-study effects in the aggregated study-level analysis. However, given the limited number of studies and the presence of between-study heterogeneity, these results should still be interpreted cautiously.

### Assessment of the quality of evidence

Using GRADEpro, we evaluated the certainty of evidence for the primary outcome, namely the effects of long-term exercise on executive function in children and adolescents with ADHD. Because all included studies were RCTs, the evidence was initially rated as high certainty. Regarding risk of bias, only four of the 15 RCTs were judged to be at low risk, whereas seven were rated as having some concerns and four as being at high risk, with the main issues arising from the randomisation process and selective reporting; therefore, the certainty of evidence was downgraded by one level for risk of bias. With respect to inconsistency, the three-level meta-analysis showed significant between-study variance (Level 3) (LRT = 25.46; *P* < 0.001), and between-study variance accounted for 48.68% of the total variance, indicating substantial heterogeneity across studies. In addition, the 95% PI was wide and crossed the null value, suggesting considerable variability in the true effects across studies. Accordingly, the evidence was downgraded by one further level for inconsistency. We did not apply a downgrade for indirectness, as the study populations, interventions, control conditions, and outcome measures were well aligned with the review question. Likewise, we did not make a downgrade for imprecision, because although the number of included studies was relatively limited, the 95% CIs of the primary pooled estimate and the sensitivity analyses did not cross the null value, and the direction and magnitude of the effect were generally consistent. We also did not apply a downgrade for publication bias, as visual inspection of the funnel plot did not suggest marked asymmetry, Egger’s regression test was not statistically significant (*t* = −1.32; *P* = 0.211), and the trim-and-fill analysis did not identify any potentially missing studies; moreover, the adjusted pooled effect remained unchanged. Overall, the certainty of evidence for the beneficial effects of long-term exercise on executive function in children and adolescents with ADHD was rated as low. This suggests that long-term exercise may have a positive effect on executive function in this population. However, the findings should be interpreted with caution because of methodological limitations in the included studies and the substantial heterogeneity observed across studies.

## DISCUSSION

We found that long-term exercise may improve executive function in children and adolescents with ADHD. This is consistent with previous research findings. A meta-analysis by Sun *et al.* demonstrated that long-term exercise can improve executive function in ADHD patients [13]. Similarly, Yang *et al.* found that aerobic exercise can enhance inhibitory control, working memory, and cognitive flexibility in children with ADHD [39]. The improvement in executive function in children and adolescents with ADHD through exercise may be related to exercise-induced changes in brain structure and function. Abnormalities in the structure and function of brain regions associated with executive function, such as the prefrontal cortex and subcortical structures, are observed in children and adolescents with ADHD. Additionally, abnormalities in functional connectivity within the fronto-striatal and fronto-parietal networks have been reported [40]. Exercise has been shown to activate brain regions related to executive function, such as the dorsolateral and ventrolateral prefrontal cortex, anterior cingulate cortex, and parietal cortex, and can enhance functional connectivity within the prefrontal executive network, including the frontal, posterior, and temporal cortical regions [41]. These effects may play a facilitating role in improving executive function in children and adolescents with ADHD.

We found that subcomponents of executive function were not identified as significant moderating factors in the improvement of executive function in children and adolescents with ADHD through exercise. Additionally, exercise showed a small to moderate effect on inhibitory control (g = −0.45), and moderate effects on working memory (g = −0.59) and cognitive flexibility (g = −0.59). Similar conclusions have been drawn in previous studies. A meta-analysis by Liang *et al.* also indicated that exercise could improve inhibitory control, working memory, and cognitive flexibility in children and adolescents with ADHD [14]. Similarly, a meta-analysis on aerobic exercise by Yang *et al.* yielded the same result [39]. However, other studies have reported different conclusions. The umbrella review by Dastamooz *et al.* suggested that exercise did not improve working memory in children and adolescents with ADHD [12]. This discrepancy may be because we included effect sizes related to working memory in a three-level meta-analysis, while Dastamooz *et al.* conducted traditional meta-analyses, which did not include all effect sizes for working memory, thus reducing statistical efficiency. In addition, we identified age as a significant moderator in the exploratory moderator analyses. Exercise showed a small effect (g = −0.40) in the <12 years subgroup and a large effect (g = −1.48) in the ≥12 years subgroup. This pattern is broadly consistent with previous research [42]. However, the ≥12 years subgroup was represented by only four effect sizes; therefore, the age-related finding should be interpreted cautiously and regarded as preliminary rather than definitive. Accordingly, age may be a potential factor to consider when designing future exercise interventions for executive function in ADHD. However, this pattern requires further confirmation in studies with better between-study replication.

We further classified outcome type into accuracy/error-based and latency-based outcomes to examine whether the effects of long-term exercise differed by outcome type. The results showed that outcome type did not serve as a significant moderator. Moreover, long-term exercise significantly improved both accuracy/error-based (g = −0.48) and latency-based outcomes (g = −0.59). These findings suggest that exercise may improve both correctness-related performance and time-related performance in executive function tasks. A recent systematic review and meta-analysis of school-aged children with ADHD suggested that differences in measurement paradigms and scoring methods may influence the estimated effects of exercise on executive function, highlighting the importance of considering measurement properties when interpreting executive function outcomes [43]. In addition, Heitz’s review noted that the speed-accuracy trade-off is a common behavioural phenomenon in cognitive tasks, indicating that response speed and response accuracy are not fully equivalent and may constrain one another [44]. Therefore, the additional classification of outcomes according to their measurement properties in the present study may help reduce interpretive bias caused by combining different types of indicators. Meanwhile, Hung *et al.* found in a task-switching study of children with ADHD that behavioural performance was often reflected simultaneously in multiple dimensions, such as longer reaction times and lower accuracy, suggesting that speed-based and accuracy-based indicators are related yet relatively distinct aspects of executive function assessment [45]. Taken together, our findings that exercise significantly improved both types of outcomes, while outcome type was not a significant moderator, suggest that the beneficial effects were relatively consistent across these two measurement dimensions rather than being driven by only one type of indicator. Future studies on exercise interventions targeting executive function in children and adolescents with ADHD should consider including both accuracy/error-based and latency-based indicators to provide a more comprehensive evaluation of intervention effects.

In the characteristics of exercise interventions, we found that exercise type, exercise frequency, and session duration were not moderators. We found open-skills exercises to improve executive function in children and adolescents with ADHD, with a moderate effect size (g = −0.59), while closed-skills exercises showed no significant effects. Similar results have been reported in previous studies. Song *et al.* concluded that closed-skills exercises did not improve inhibitory control, working memory, or cognitive flexibility in children and adolescents with ADHD, while open-skills exercises improved executive function in this population [38]. However, Huang *et al.* suggested that both open- and closed-skills exercises could improve executive function in children and adolescents with ADHD [42]. Because exercise type was not identified as a significant moderator in the present study, the apparent difference between open- and closed-skill exercise should not be overinterpreted as evidence that one exercise type is definitively superior to the other. Rather, our findings suggest that open-skill exercise shows promise, while the evidence for closed-skill exercise remains less stable and may require further investigation. Regarding exercise frequency, we observed that exercise performed 2–3 times per week was associated with a significant pooled effect, whereas the 4–5 times per week subgroup did not reach statistical significance. However, because exercise frequency was not a significant moderator, this finding should not be interpreted as evidence that 2–3 sessions per week is necessarily more effective than 4–5 sessions per week. This may be because higher frequency interventions may result in lower adherence and motivation among children and adolescents [46]. Session duration was also not a moderator. Interventions of >60 minutes had a large effect size (g = −1.0), while interventions of ≤60 minutes showed a small-to-moderate effect size (g = −0.42). The study by Huang *et al.* also indicated that interventions lasting >50 minutes had better effects [42]. This may be because longer interventions are more effective in enhancing functional connectivity between brain regions, thereby promoting improvements in executive function. Some studies have also shown that moderate-intensity exercise for 50 minutes is more beneficial for brain functional connectivity in children with ADHD compared to 30 minutes of exercise [15]. Additionally, Yang *et al.* found that both 15–50 minutes and 60–90 minutes of exercise improved executive function in children and adolescents with ADHD, with the latter showing better effects. This may be because we included all types of exercise, while Yang *et al.* focused only on aerobic exercise. Nevertheless, because session duration was not a significant moderator, these subgroup differences should be interpreted cautiously and should not be taken as firm evidence that longer sessions are superior.

Moreover, we found that intervention duration was identified as a significant moderator in the exploratory analyses of the effect of exercise interventions on executive function in children and adolescents with ADHD. Specifically, intervention duration lasting >12 weeks showed a large effect size (g = −1.40), whereas interventions lasting 6–12 weeks showed a small-to-moderate effect size (g = −0.40). This pattern may suggest that longer intervention cycles are associated with larger benefits. Long-term exercise has been shown to enhance the functional connectivity and overall efficiency of neural networks, particularly in brain regions associated with executive function, such as the anterior cingulate cortex, occipital lobe, and frontal lobe. Additionally, long-term exercise has been shown to increase neuronal nutrient supply in the hippocampus, which may also contribute to the improvement of executive function [47,48]. To further explore the dose-response relationship of exercise interventions, we also examined weekly exercise time (session duration × weekly frequency) and total exercise time (weekly exercise time × intervention duration) as continuous moderators. The results showed that weekly exercise time was not significantly associated with improvements in executive function in either the linear or nonlinear models, suggesting that simply increasing the amount of exercise performed per week may not necessarily lead to greater benefits. One possible explanation is that weekly exercise time reflects a composite indicator of session duration and frequency, and its effect may be influenced by multiple factors such as adherence, fatigue, and individual tolerance, thereby obscuring a clear dose–response pattern. By contrast, total exercise time showed a marginally significant linear trend, with larger cumulative exercise exposure tending to be associated with greater improvements in executive function. However, the overall nonlinear model for total exercise time was not statistically significant, indicating a lack of evidence to support a clear nonlinear dose-response relationship or a specific threshold effect. Taken together, these findings suggest that cumulative exercise exposure may be relevant to executive function improvement in children and adolescents with ADHD, but the current evidence remains preliminary and should be interpreted with caution. However, Yang *et al.* demonstrated that exercise lasting 6–12 weeks can also improve executive function. This discrepancy may arise because we used a three-level meta-analysis approach, which included all relevant effect sizes, while Yang *et al.* included only a subset of effect sizes. Furthermore, we incorporated all types of exercise, including aerobic and resistance training, while Yang *et al.* focused only on aerobic exercise [39]. Importantly, the >12 weeks subgroup was supported by only four effect sizes. Therefore, the relatively large pooled effect in this subgroup should be interpreted cautiously, as it may partly reflect study-specific characteristics rather than a stable duration-response pattern. Accordingly, the intervention-length finding is better regarded as exploratory and requires confirmation in future studies. Additionally, differences in the severity of ADHD, disease duration, and the extent of executive function impairments in the populations studied may have introduced some bias in the results, which warrants further investigation in future studies.

### Study strengths and limitations

Our study has several strengths. We focused on RCTs, which strengthens the internal validity and overall robustness of our findings. Also, we included all relevant effect sizes related to executive function and employed a three-level meta-analysis to account for the dependency among multiple effect sizes within studies, thereby improving statistical efficiency and yielding more robust estimates. Additionally, we explored several potential moderators of the effects of exercise on executive function in children and adolescents with ADHD, which may help to identify possible sources of heterogeneity.

Our study also has several limitations. First, although we explored a range of potential moderators, the categorisation of some variables was relatively coarse, and several subgroup strata were supported by only a limited number of effect sizes, particularly the >12 weeks and ≥12 years categories. In addition, because some of the included studies reported insufficient intervention details, we could not examine certain potentially important moderators, such as exercise intensity. Therefore, the moderator findings should be regarded as exploratory and interpreted with caution. Second, we only included studies published in Chinese and English, which may have introduced language bias and potentially increased the risk of publication bias. Third, we assessed the outcome indicators using different measurement tools with varying reliability and sensitivity, potentially reducing the comparability and credibility of the findings. Moreover, in some studies, the SDs of change scores were not directly reported, so we derived them from the baseline and post-intervention SDs using an assumed pre-post correlation coefficient. Although this approach allowed for a consistent calculation of effect sizes across studies, the assumed correlation may still have introduced some uncertainty. Fourth, publication-bias-related analyses should be interpreted cautiously. Although we conducted funnel plot inspection, Egger’s regression test, and trim-and-fill analyses as supplementary assessments based on study-level aggregated effect sizes, these methods may perform suboptimally in the presence of substantial heterogeneity, a limited number of studies, and multiple dependent effect sizes within studies. In addition, medication status was inconsistently reported across the included studies, and several studies did not provide sufficient information on whether ADHD medication use was controlled or balanced at baseline. As stimulant medication may affect executive function outcomes and adherence to exercise interventions, this unmeasured clinical heterogeneity may also have contributed to the between-study variance. Therefore, the overall findings should be interpreted with caution.

### Prospects for future research

Based on the gaps and limitations identified in the currently available evidence, we propose the following directions for future research. First, future studies should clearly define all relevant aspects of the experimental protocol, such as exercise intensity. Additionally, further exploration of various exercise modalities, such as yoga and tai chi, is warranted to identify more suitable exercise programmes for individuals with ADHD. Second, future research should not only assess the effectiveness of exercise interventions in children and adolescents with ADHD, but also explore the physiological and neurological mechanisms underlying these effects. Third, future studies should expand sample sizes and improve between-study replication within key subgroup strata, especially for age and intervention-length categories, so that moderator findings can be evaluated more robustly. Lastly, future studies should report both correctness-related and time-related executive function outcomes whenever possible, to reduce interpretive bias related to speed-accuracy trade-offs and to facilitate more refined evidence synthesis.

## CONCLUSIONS

Long-term exercise may be beneficial for executive function in children and adolescents with ADHD, although the certainty of the evidence was low. In addition, findings related to age and intervention duration should be interpreted with caution, as some key subgroup strata were based on only a small number of effect sizes. These moderator findings are therefore better regarded as exploratory and should be confirmed in future studies.

## Additional material


Online Supplementary Document
